# Tryptophan metabolic reprogramming modulates cytokine networks in nucleos(t)ide analogue-treated chronic hepatitis B patients

**DOI:** 10.3389/fcimb.2025.1643636

**Published:** 2025-07-21

**Authors:** Xuedan Gao, Xiaojuan Wu, Yi Li, Xinhua Dai, Bei Cai

**Affiliations:** ^1^ Department of Laboratory Medicine, West China Hospital, Sichuan University, Chengdu, China; ^2^ Laboratory Medicine Research Center of West China Hospital, Sichuan University, Chengdu, China; ^3^ Sichuan Clinical Research Center for Laboratory Medicine, Chengdu, China

**Keywords:** tryptophan metabolism, cytokine profiles, chronic hepatitis B, bacterial degradation pathway, functional cure, indole-3-carboxaldehyde, picolinic acid, 5-hydroxytryptamine

## Abstract

**Background & aims:**

Hepatitis B virus (HBV) infection remains a global health challenge. Tryptophan metabolism influences immune regulation, but its interplay with cytokines during antiviral therapy is unclear. We investigated associations between tryptophan pathways and cytokine profiles in the chronic hepatitis B (CHB) patients with varying treatment outcomes.

**Methods:**

This retrospective study included 106 CHB patients (including 29 functional cure cases) receiving nucleos(t)ide analogues (NAs) and 29 healthy controls. Plasma levels of 20 tryptophan metabolites (kynurenine, serotonin, and bacterial pathways) were quantified by HPLC-MS/MS, and 12 cytokines were measured via flow cytometry. Multivariate analyses were performed.

**Results:**

Functional cure patients showed unique metabolic patterns. Indole-3-carboxaldehyde (IAld) levels increased progressively from HBsAg positive groups (HBeAg-: 63.324 nmol/L; HBeAg+: 65.938 nmol/L) to functional cure (91.44 nmol/L) and healthy controls (130.634 nmol/L) (*P* < 0.01), exhibiting negative correlations with HBsAg (*r* = -0.31) and IFN-γ (*r* = -0.53) but positive correlation with IL-1β (*r* = 0.47). Picolinic acid (PA) was significantly elevated in the functional cure group (*P *< 0.001), associated with reduced HBsAg, IL-2 and increased IL-1β, IL-10 levels, indicating potential antiviral effects. Serotonin (5-HT) levels were higher in cured patients and correlated with IL-1β and IFN-α (*P* < 0.05). HBeAg-positive patients displayed increased kynurenine-to-tryptophan (Kyn/Trp) ratios (*P* < 0.05), while non-cured patients showed metabolic blockade downstream of 3-hydroxykynurenine (elevated 3-HK/Kyn ratios and reduced KA, XA/3-HK, 3-HAA/3-HK, and NAA levels; *P* < 0.05).

**Conclusions:**

The tryptophan metabolites (IAld, PA, 5-HT) were found to correlate with cytokine levels (IL-1β, IL-10), potentially implicating their involvement in immune regulation and antiviral responses. These observations delineate a metabolic-immune framework that may inform future therapeutic development for HBV.

## 1 Introduction

Hepatitis B Virus (HBV) infection remains a major global public health challenge and one of the leading causes of liver disease-related deaths worldwide. According to the Global Burden of Disease (GBD) 2019 study, the global prevalence of chronic HBV infection across all age groups was estimated at 4.1% (95% uncertainty interval: 3.7-4.5), corresponding to 316 million infected individuals (GBD 2019 Hepatitis B Collaborators, 2022). The World Health Organization (WHO) estimated in 2024 that about 1.23 million new HBV infections occur annually globally, and about 1.1 million people die from diseases associated with HBV infection (World Health Organization, 2024). Although the current first-line antiviral therapies, Nucleos(t)ide Analogues (NAs) and Pegylated Interferon-alpha (PEG-IFNα), are effective in suppressing viral replication and slowing the progression of cirrhosis and hepatocellular carcinoma (HCC), functional cure rates (defined as sustained HBsAg clearance) remain below 10%, and most patients require lifelong treatment or experience poor tolerability (Wu et al., 2025; Yuen et al., 2018). The key to achieving a functional cure for chronic hepatitis B (CHB) lies in suppressing HBV-DNA replication and clearance of viral antigens (HBsAg and HBeAg). However, the relationship between this process and metabolic reprogramming remains unclear.

In recent years, the application of metabolomics in liver disease research has become increasingly widespread. Li Hai’s team identified numerous potential prognostic and diagnostic biomarkers for acute-on-chronic liver failure (ACLF) through untargeted metabolite analysis, including membrane lipid metabolism, steroid hormones, oxidative stress pathways, and energy metabolism, and developed targeted liquid chromatography tandem mass spectrometry (LC-MS/MS) assays for four metabolites for clinical laboratories use (Zhang et al., 2023). Additionally, metabolic comorbidities (such as obesity and diabetes) have been shown to exacerbate liver fibrosis in HBV patients and reduce fibrosis regression rates after antiviral therapy (van Velsen et al., 2025). Tryptophan is metabolized through the kynurenine pathway, the serotonin (5-HT) pathway, and the bacterial degradation pathway, with its metabolites (such as kynurenine and indole derivatives) participating in the regulation of various pathophysiological processes, including protein synthesis, inflammation, oxidative stress, and immune responses (Xue et al., 2023). Numerous studies have shown that tryptophan metabolites modulate systemic inflammation. For instance, indoleamine 2,3-dioxygenase (IDO), an interferon-γ-induced enzyme in the tryptophan pathway, catalyzes the conversion of tryptophan to kynurenine (Werner et al., 1987) and exhibits a dual role in infectious diseases-both promoting inflammation and regulating acute and chronic infections (Mehraj and Routy, 2015). Previous studies have found that platelet-derived 5-HT exacerbates viral hepatitis (Lang et al., 2008). However, other studies suggest that 5-HT-mediated DDX37 agonists (AS-19) increase IFN-β expression and inhibit HBV replication (Kang et al., 2019).

Numerous studies have demonstrated that cytokine networks dictate the immunopathology of hepatitis B virus infection. For instance, Th1 cytokines (e.g., IFN-γ, IL-2) promote viral clearance (Li et al., 2016), whereas in chronic viral infections, IL-10 frequently induces T cell exhaustion and inactivates antiviral T-cell immunity by modulating T cells and antigen-presenting cells (APCs), thereby facilitating immune evasion and persistent/latent infection (Rojas et al., 2017). Recent evidence indicates that tryptophan metabolites modulate cytokine responses. For example, IDO increases populations of T lymphocytes producing IFN-γ and IL-17, thereby exerting inhibitory effects (Huang et al., 2020). Indole-3-carboxaldehyde (IAld) restores virus-induced pro-inflammatory features by binding to the aryl hydrocarbon receptor (AhR), reducing IL-1β production and increasing IL-10 (Pariano et al., 2024). Additionally, serotonin stimulates secretion of pro-inflammatory cytokines (IL-1 and IL-6) while enhancing the cytotoxicity of IFN-γ, playing a significant role in antiviral defense (Kanova and Kohout, 2021).

However, research on the tryptophan metabolic pathway related to HBV remains limited. As a key immunoregulatory metabolic pathway, its role in viral antigen clearance during chronic HBV infection has yet to be elucidated. Therefore, this study focuses on patients with different CHB disease status, aiming to investigate the relationship between tryptophan metabolites and viral antigen clearance. The findings may reveal the immune regulatory function of tryptophan metabolism in HBV and provide novel therapeutic targets for optimizing clinical cure strategies.

## 2 Materials and methods

### 2.1 Study population

This retrospective analysis included 135 participants: 106 CHB patients (including functional cure cases) who received NAs therapy for 48 weeks (without prior interferon treatment) and were followed at our outpatient clinic from July 2023 to August 2024, along with 29 healthy controls. All patients met the diagnostic criteria before treatment outlined in the Chinese *Guidelines for the Prevention and Treatment of Chronic Hepatitis B (2022 Edition)* (Chinese Society of Hepatology, C.M.A and Chinese Society of Infectious Diseases, Chinese Medical Association, 2022). And functional cure was defined according to the *Expert Consensus on Clinical Cure (Functional Cure) of Chronic Hepatitis B* (Chinese Society of Infectious Disease Chinese Society of Hepatology, C.M.A, 2019), characterized as sustained, undetectable serum HBsAg, HBeAg and HBV DNA with or without seroconversion to anti-HBs, but with the persistence of residual cccDNA, accompanied by resolution of liver injury after the completion of a finite course of treatment. Healthy controls were selected from individuals undergoing routine health examinations at our center. Exclusion criteria included acute hepatitis B, severe hepatitis B, overlapping-infections (other viruses/bacteria), autoimmune diseases, liver cirrhosis, and malignancies. Baseline clinical and laboratory characteristics are summarized in **Table 1**. This study was approved by the Ethics Committee of West China Hospital of Sichuan University (No: 2021-140). The sample used in this study were residual blood samples from clinical testing, and informed consent from patients was waived.

**Table 1:** Baseline clinical characteristics and laboratory parameters.

Group^a^	Functional cure (n = 29)	HBeAg-negative (n = 40)	HBeAg-positive (n = 37)	Healthy Control (n = 29)	Total (n = 135)	*P value^c^*
Age (y)	48.38 ± 12.77	45.88 ± 9.49	39.54 ± 9.03	43.38 ± 10.92	44.14 ± 10.86	0.006
Sex (n (%))						
Male	26 (89.7%)	20 (50%)	22 (59.5%)	11 (37.9%)	79 (58.5%)	< 0.001
Famale	3 (10.3%)	20 (50%)	15 (40.5%)	18 (62.1%)	56 (41.5%)	
Disease duration (y) ^b^						
<5	1 (4.2%)	5 (15.2%)	4 (13.3%)	/	10 (11.5%)	0.382
5~10	2 (8.3%)	7 (21.2%)	3 (10%)	/	12 (13.8%)	
10~20	11 (45.8%)	12 (36.4%)	16 (53.3%)	/	39 (44.8%)	
>20	10 (41.7%)	9 (27.3%)	7 (23.3%)	/	26 (29.9%)	
Duration of treatment (y)	5 (3, 10)	3 (1, 7)	5 (2.25, 8)	/	5 (2, 8)	0.085
HBsAg (IU/mL)	/	1091.5 (383.75, 2901)	1805 (777, 8898)	/	/	/
HBV DNA (n (%))						
<100 copies/mL	29 (100%)	40 (100%)	33 (89.2%)	/	102 (96.2%)	0.017
≥100 copies/mL	/	/	4 (10.8%)	/	4 (3.8%)	
ALT (U/L)	26 (16, 34)	23 (17, 31)	22 (15.5, 34.5)	15.5 (9, 21.25)	22 (15, 29)	0.002
AST (U/L)	24 (21, 25)	23 (21, 27)	23 (20, 26)	17.5 (15, 21)	22 (18, 25)	< 0.001
ALP (IU/L)	83.81 ± 16.38	83.36 ± 25.16	79.08 ± 20.30	63.24 ± 15.86	77.79 ± 21.66	< 0.001
GGT (IU/L)	22 (14, 29)	18 (14, 28)	20 (12, 32)	15 (10, 19.25)	17 (12, 26)	0.045
ALB (g/L)	48.50 ± 2.39	47.97 ± 2.42	47.25 ± 2.24	46.10 ± 2.54	47.46 ± 2.51	0.001
WBC (×10^9^/L)	5.84 ± 1.62	5.62 ± 1.26	6.22 ± 1.43	5.23 ± 1.11	5.74 ± 1.38	0.031
AFP (ng/mL)	2.44 (1.65, 3.18)	2.51 (1.78, 3.53)	2.49 (1.92, 3.52)	2.35 (2.04, 4.01)	2.46 (1.87, 3.44)	0.638

a Functional cure: HBV-infected patients with sustained HBsAg loss and undetectable HBV DNA; HBeAg-negative: patients with HBsAg+HBeAg-HBcAb+; HBeAg-positive: patients with HBsAg+HBeAg+HBcAb+.

b Relevant data were missing for 19 patients.

c *P* value < 0.05 indicates statistical significance.

ALT, Alanine Aminotransferase; AST, Aspartate Aminotransferase; ALP, Alkaline Phosphatase; GGT, Gamma-Glutamyl Transferase; ALB, Albumin; WBC, White Blood Cell Count; AFP, Alpha-Fetoprotein.

### 2.2 HPLC-MS/MS analysis of tryptophan and its metabolites

High-performance liquid chromatography-mass spectrometry (HPLC-MS/MS) was used to quantitatively detect tryptophan and its metabolites in plasma, covering 20 metabolites across three pathways (**Supplementary Figure 1**):

- Kynurenine pathway: Tryptophan (Trp), Kynurenine (Kyn), 3-Hydroxykynurenine (3-HK), Kynurenic acid (KA), Xanthurenic acid (XA), 3-Hydroxyanthranilic acid (3-HAA), Quinolinic acid (QA), Picolinic acid (PA), Nicotinic acid (NA), Nicotinamide (NAA).
- Serotonin (5-HT) pathway: 5-Hydroxytryptophan (5-HTP), 5-Hydroxytryptamine (5-HT) (Serotonin), 5-Hydroxyindole-3-acetic acid (5-HIAA), N-Acetylserotonin (NAS), Melatonin (M).
- Bacterial degradation pathway (Indole pathway): Tryptamine (TA), IAld, indole-3-acetic acid (IAA), Indole-3-lactic acid (ILA), Indolepropionic acid (IPA).

The analytical methods were referenced from previous studies by our research group (Miao et al., 2025). Additionally, this study incorporated several calculated values to enhance the analytical strategy, such as Sum-5-HT, Sum-Indoles, and Sum-Kyn, which represent the overall levels of all metabolites in the 5-HT pathway, bacterial degradation pathway, and kynurenine pathway, respectively. Furthermore, metabolite-to-precursor ratios were used, such as Kyn/Trp, KA/Kyn, 3-HK/Kyn, XA/3-HK, 3-HAA/3-HK, QA/3-HAA, and PA/3-HAA, which reflect the activity of key enzymes in metabolic processes (Moulin et al., 2024).

### 2.3 Flow cytometry assay for cytokines

Plasma cytokine profiling was performed using FACS Canto II ™ flow cytometer (BD Biosciences, Franklin Lakes, NJ, USA) with a multiplex cytokine assay kit (immunofluorescence method) (Jiangxi Saiji Biotechnology Co., Ltd., Nanchang, China) according to the manufacturer’s protocol. Twelve cytokines were simultaneously quantified: IL-2, IL-4, IL-6, IL-10, TNF-α, IFN-γ, IL-17, IL-1β, IL-5, IL-12p70, IFN-α, and IL-8.

### 2.4 Statistical analysis

Statistical analyses were performed using SPSS 27.0 (IBM SPSS Software Inc., Armonk, NY, USA) and figures were produced with Origin 2024 (OriginLab Corporation, Northampton, MA, USA) including principal component analysis, heatmaps, and violin plots. Figure of tryptophan metabolic pathway was designed using Microsoft PowerPoint (Microsoft Corporation, Redmond, WA, USA). All clinical characteristics of patients were analyzed using descriptive statistics. For continuous variables with a normal distribution, data were expressed as mean ± standard deviation (Mean ± SD). For continuous variables with a non-normal distribution, data were expressed as median (interquartile range) (Median (P25, P75)). For categorical variables, data were expressed as frequency (percentage) (n (%)). Group comparisons were conducted using one-way ANOVA for normally distributed continuous variables, Kruskal-Wallis H test for non-normally distributed continuous variables, and chi-square test for categorical variables. Both univariate and multivariate logistic regression analyses were conducted to control for potential confounding factors. Spearman’s rank correlation was used to evaluate relationships between metabolite levels and cytokine levels. A *P*-value < 0.05 was considered statistically significant.

## 3 Results

### 3.1 Clinical characteristics of the study cohort

This study enrolled 106 patients with CHB who received NAs therapy for over 48 weeks, stratified into four groups based on treatment outcomes: Functional cure group (n = 29), HBeAg-negative (HBsAg+HBeAg-HBcAb+) group (n = 40), HBeAg-positive (HBsAg+HBeAg+HBcAb+) group (n = 37), and Healthy control group (n = 29). As summarized in **Table 1**, the overall age of participants was 44.14 ± 10.86 years, with the functional cure group demonstrating the highest mean age (48.38 ± 12.77 years), while the HBeAg-negative group and HBeAg-positive group had relatively lower mean ages (45.88 ± 9.49 years and 39.54 ± 9.03 years, respectively). The proportion of males in the functional cure group (89.7%) were significantly higher than that in the HBeAg-positive group (50%) and HBeAg-negative group (59.5%). While disease duration and treatment length varied considerably across patient groups, no significant intergroup differences were observed. Laboratory analyses showed that liver function parameters (Alanine aminotransferase (ALT), Aspartate aminotransferase (AST), Alkaline phosphatase (ALP), Gamma-glutamyl transferase (GGT), Albumin (ALB)) were higher in all disease groups than in the healthy control group. White blood cell count (WBC) was highest in the HBeAg-positive group and lowest in controls. And there were no statistically significant differences in alpha-fetoprotein (AFP) levels between groups.

The baseline imbalances in laboratory indicators were attributed to differences between the disease group and healthy controls, while no significant differences were observed between functional cure and non-cured patients within the disease group (**Supplementary Table 1**). Therefore, we adjusted only for confounding factors including age and sex in both univariate and multivariate regression analyses (**Table 2**). Univariate logistic regression analysis revealed that multiple tryptophan metabolic pathway markers and cytokines were significantly associated with functional cure in chronic hepatitis B (CHB) patients. Subsequently, multivariate logistic regression analysis using the forward likelihood ratio (Forward: LR) method identified IAld, PA, PA/3-HAA ratio, and TNF-α as independent predictors.

**Table 2:** Logistic regression analysis of tryptophan metabolites and cytokines on the functional cure of hepatitis B^a^.

Variables	Univariate analysis		Multivariate analysis^b^	
	OR(95% CI)	*P* value^c^	OR(95% CI)	*P* value^c^
Age (y)	0.954 (0.916, 0.993)	0.023	NA	0.144
Sex (male)	1.131 (0.037, 0.471)	0.002	NA	0.084
Sum-5HT (nmol/L)	0.994 (0.99, 0.999)	0.010	NA	0.208
5-HT (nmol/L)	0.994 (0.989, 0.999)	0.011	NA	0.207
IAlD (nmol/L)	0.98 (0.968, 0.993)	0.002	0.94 (0.906, 0.975)	0.001
Sum-Kyn (nmol/L)	0.999 (0.999, 1)	0.036	NA	0.867
Kyn (nmol/L)	0.999 (0.998, 1)	0.024	NA	0.619
PA (nmol/L)	0.878 (0.83, 0.929)	0.000	0.909 (0.835, 0.991)	0.030
Kyn/Trp	0.919 (0.859, 0.984)	0.015	NA	0.977
XA/3-HK	0.053 (0.003, 0.972)	0.048	NA	0.713
PA/3-HAA	0.412 (0.277, 0.615)	0.000	0.371 (0.171, 0.806)	0.012
IL-2 (pg/mL)	2.545 (1.446, 4.479)	0.001	NA	0.171
IL-10 (pg/mL)	0.586 (0.451, 0.761)	0.000	NA	0.988
TNF-α (pg/mL)	0.58 (0.453, 0.744)	0.000	0.361 (0.205, 0.638)	< 0.001
IL-8 (pg/mL)	0.913 (0.839, 0.993)	0.034	NA	0.190
IL-6/IL-10	7.697 (1.379, 42.951)	0.020	NA	0.962

a The dependent variable is a binary classification variable (functional cure (n = 29) and non-cured (HBeAg-negative and HBeAg-positive (n = 77)).

b The final model was determined through multivariate analysis using the Forward: LR method, with collinearity verified by VIF (all variables had VIF < 5). NA, ORs cannot be calculated for variables not retained in the final model equation.

c *P* value < 0.05 indicates statistical significance.

OR, Odds Ratio; CI, confidence interval; Sum-5-HT, Collective metabolites in the serotonin pathway; 5-HT, 5-Hydroxytryptamine (Serotonin); NAS, N-Acetylserotonin; IAlD, Indole-3-carboxaldehyde; Sum-Kyn, Collective metabolites in the kynurenine pathway; Kyn, Kynurenine; PA, Picolinic acid; Kyn/Trp, ratio of Kynurenine to Tryptophan; XA/3-HK, ratio of Xanthurenic acid to 3-Hydroxykynurenine; PA/3-HAA, ratio of Picolinic acid to 3-Hydroxyanthranilic acid; IL, Interleukin; TNF, Tumor Necrosis Factor.

### 3.2 Analysis of tryptophan and its metabolites levels

Comprehensive profiling of all metabolites and calculated indices was performed, with NA and TA metabolites excluded from detailed analysis due to their consistently low concentrations across all groups. The principal component analysis (PCA) was performed on the combined dataset of tryptophan metabolites and cytokines from all four study groups (**Supplementary Figure 2**). Principal components 1 and 2 accounted for 20.8% and 12.3% of the total variance, respectively. Distinct clustering was observed between all patient groups and healthy controls, whereas the HBeAg-negative and -positive groups demonstrated overlapping distributions. Additional PCA analyses stratifying the functional cure group and non-cured subgroups are presented in **Supplementary Figure 3** (which includes separate PCA analyses for metabolites and cytokines).

Our findings demonstrated statistically significant differences (*P* < 0.05) in multiple metabolic pathways among groups (**Supplementary Table 2**), including Trp and Sum-5-HT, 5-HT, NAS, M in the 5-HT pathway, IAld and ILA in the bacterial degradation pathway, and Sum-Kyn, KA, PA, NAA, Kyn/Trp, 3-HK/Kyn, XA/3-HK, 3-HAA/3-HK, and PA/3-HAA in the kynurenine pathway.

**Figure 1:**
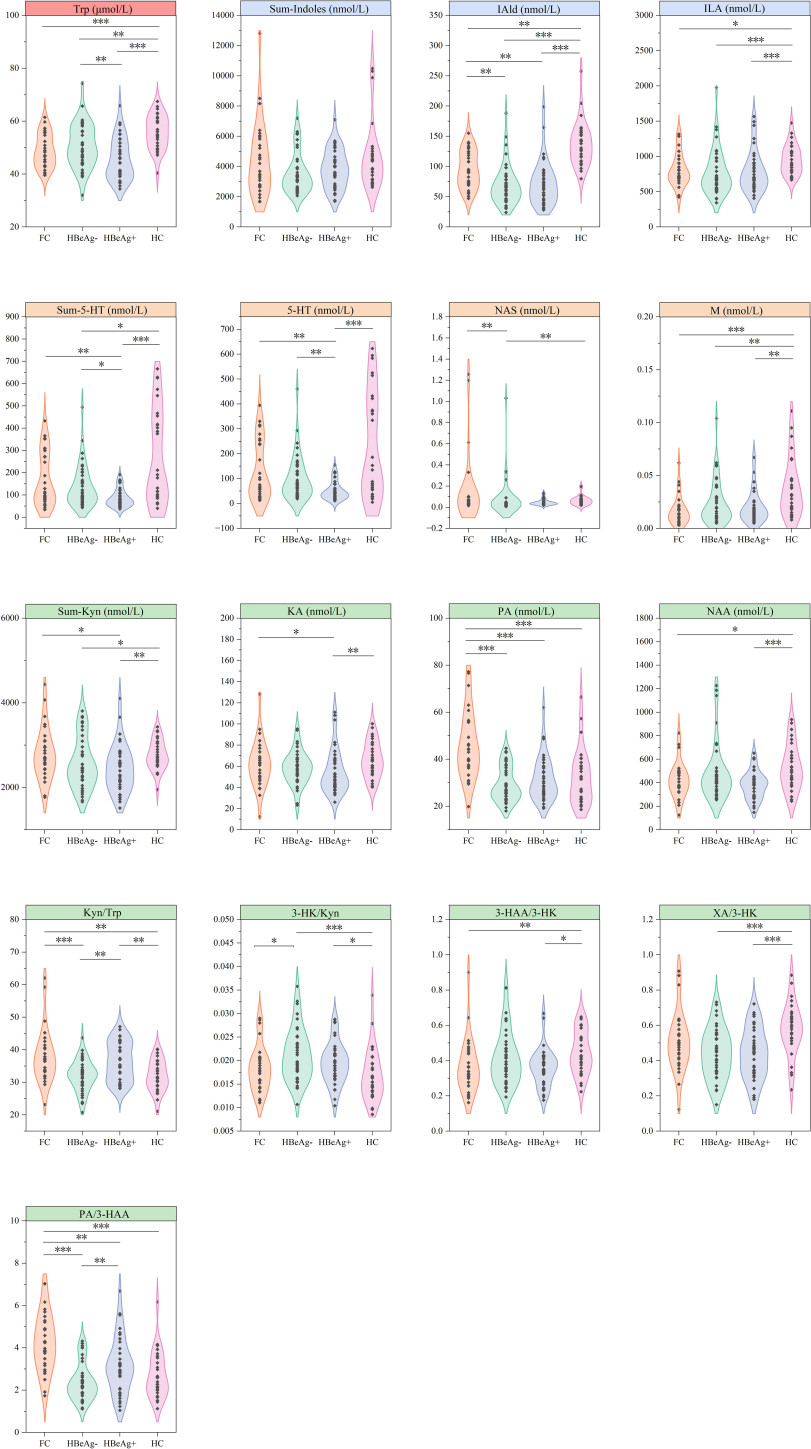
Differential analysis of tryptophan metabolites across groups. FC, Functional cure group (n = 29); HBeAg-, HBeAg-negative group (n = 40); HBeAg+, HBeAg-positive group (n = 37); HC, Healthy Control group (n = 29). *P < 0.05, **P < 0.01, ***P < 0.001.

For metabolites and calculated values with significant differences, we further conducted intergroup differential analysis (**Figure 1**). The results showed significantly elevated tryptophan levels in healthy controls compared to all patient groups, with the HBeAg-positive cohort exhibiting obvious reduction in tryptophan concentration. Which indicating that tryptophan metabolism may be inhibited in HBV patients and is closely related to disease efficacy.

In the downstream bacterial degradation pathway of tryptophan, IAld and ILA were also significantly higher in the healthy control than in the disease groups, while IAld exhibited a progressively higher levels from HBsAg-positive (median 63.324 nmol/L in HBeAg-negative and 65.938 nmol/L in HBeAg-positive) to functional cure groups (91.44 nmol/L) and finally to the healthy control groups (130.634 nmol/L, *P* < 0.01), showing a gradual increase as HBV antigen levels decreased.

Similar patterns were observed in the 5-HT pathway, with Sum-5-HT and 5-HT exhibiting comparable variations across groups, with no significant differences between the healthy control group and the functional cure group but were markedly higher than non-cured HBV groups, particularly the HBeAg-positive cohort. Although there were no significant differences between the functional cure group and the HBeAg-negative group, both were significantly higher than the HBeAg-positive group. The two metabolites NAS and M in this pathway also showed higher levels in the healthy control group than in the disease group.

The kynurenine pathway revealed the most differential metabolites and indices, with the overall metabolite level Sum-Kyn showing no significant difference between healthy controls and the functional cure group, but both were significantly higher than non-cured groups. While the functional cure and HBeAg-negative groups showed comparable levels, they differed significantly from the HBeAg-positive group. Analysis of downstream kynurenine metabolites and metabolic enzymes revealed that while Kyn/Trp (reflecting Indoleamine 2,3-dioxygenase (IDO) activity) exhibited unclear variations across groups, the remaining differential metabolites consistently demonstrated significant differences between patient groups and healthy controls. Specifically, 3-HK/Kyn (indicating kynurenine 3-monooxygenase (KMO) activity) was elevated in non-cured groups, whereas KA (the metabolic product of Kyn and kynurenine aminotransferase (KAT)), XA/3-HK (representing KAT II activity), 3-HAA/3-HK (reflecting kynureninase (KYNU) activity), and NAA (the terminal product of kynurenine pathway) were all reduced in patient groups. These observations suggest that NAs-treated HBV patients, particularly those who failed to achieve functional cure, may experience metabolic inhibition downstream of 3-HK in the kynurenine pathway. Interestingly, PA, products of another metabolic pathway of 3-HAA, and PA/3-HAA, were significantly elevated only in the cured group.

These findings suggests that all three tryptophan metabolic pathways exhibit meaningful changes following NAs therapy in HBV patients.

### 3.3 Cytokine profile analysis

**Figure 2:**
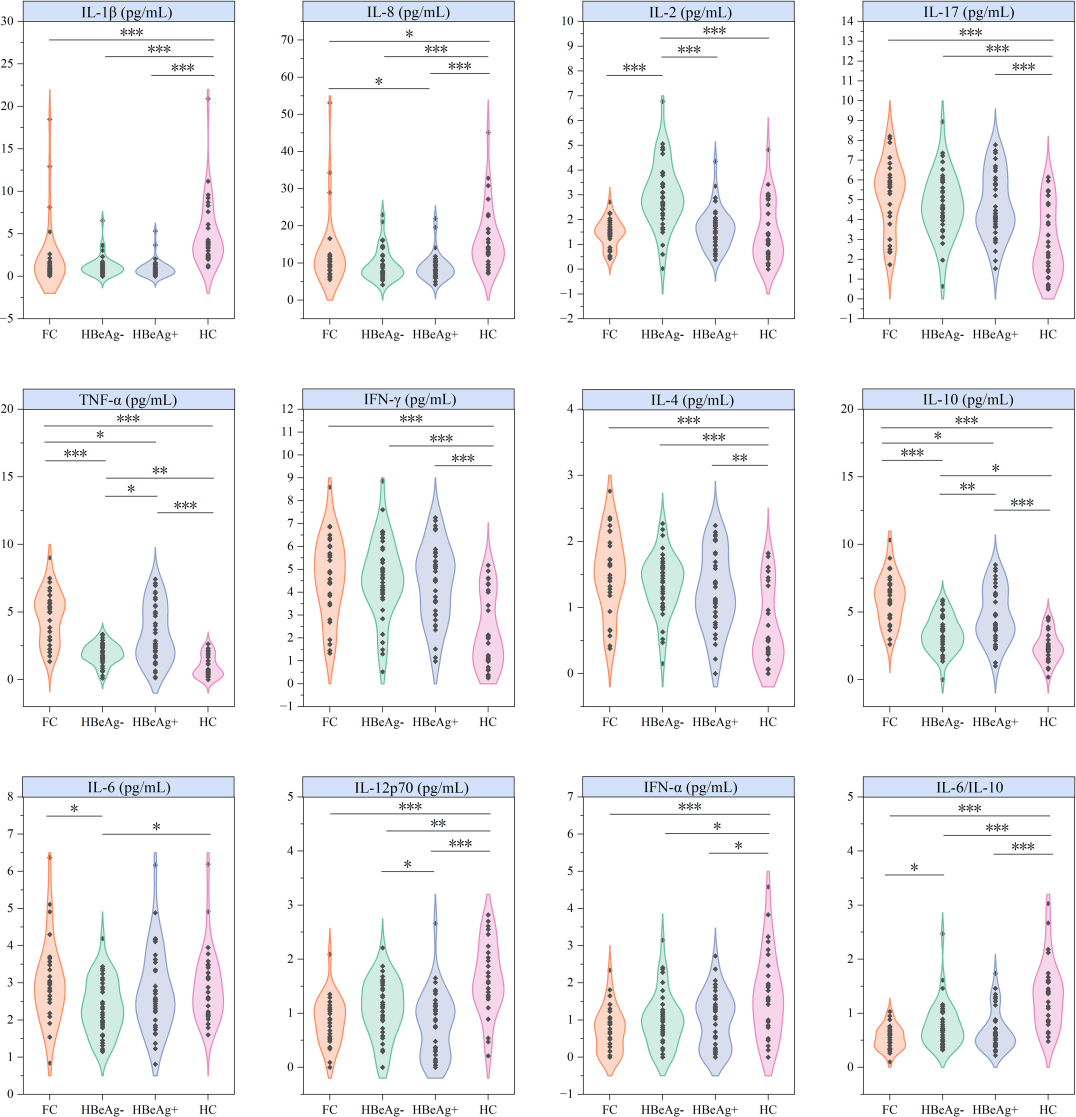
Differential analysis of cytokines across groups. FC, Functional cure group (n = 29); HBeAg-, HBeAg-negative group (n = 40); HBeAg+, HBeAg-positive group (n = 37); HC, Healthy Control group (n = 29). *P < 0.05, **P < 0.01, ***P < 0.001.

In the intergroup comparison of cytokines, all cytokines except IL-5 showed significant differences between different groups (**Figure 2**), demonstrating persistent immune dysregulation in both HBV-infected patients and those achieving clinical cure. Specifically, healthy controls showed markedly higher levels of IL-1β, IL-8, IL-12p70, IFN-α, and IL-6/IL-10 ratio compared to patient groups, while exhibiting significantly lower concentrations of IL-17, TNF-α, IFN-γ, IL-4, and IL-10. Notably, the HBeAg-negative group displayed distinct cytokine patterns characterized by elevated IL-2 levels, reduced TNF-α and IL-10 concentrations, and lower IL-6 levels compared to both functional cure patients and healthy controls.

### 3.4 Correlation analysis between tryptophan metabolites and cytokine levels

**Figure 3:**
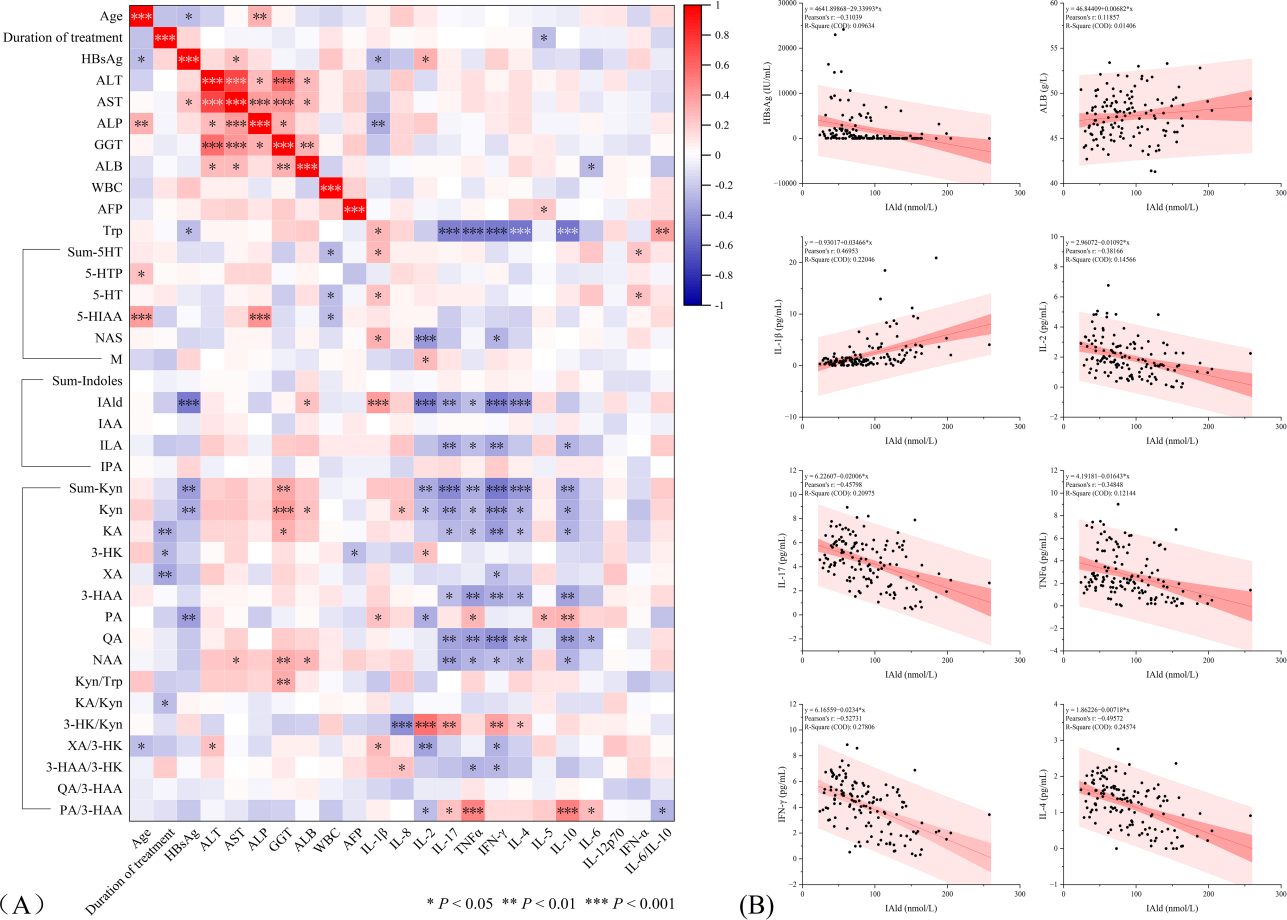
**(A)** Correlation between tryptophan metabolites and cytokines in NAs-treated HBV patients. The correlations with statistical significance are marked with asterisks (*). **(B)** Linear regression plots of parameters significantly associated with IAld. *P < 0.05, **P < 0.01, ***P < 0.001.

We examined correlations between patient basic information and important laboratory parameters with tryptophan metabolites and cytokines. The heatmap shows the strength of correlation between each indicator, with statistically significant correlations indicated by *P* values (**Figure 3A**). The results showed significant correlations between multiple tryptophan metabolites and cytokines. Overall, Trp, bacterial degradation pathway metabolites (IAld and ILA), and kynurenine pathway metabolites (Sum-Kyn, Kyn, KA, 3-HAA, QA, and NAA) were significantly negatively correlated with IL-2, IL-17, TNF-α, IFN-γ, IL-4, and IL-10. Sum-5-HT, 5-HT, and NAS in the 5-HT pathway were positively correlated with IL-1β and IFN-α. As mentioned earlier, we observed that IAld exhibited a satisfactory gradient across all subgroups, and IAld also showed a statistically significant correlation with HBsAg. The observed patterns indicate this substance could be clinically significant in treatment response. We therefore generated linear regression plots specifically for IAld (**Figure 3B**), revealing a weak positive correlation with ALB (*r* = 0.11), moderate positive correlation with IL-1β (*r* = 0.47), weak negative correlation with HBsAg (*r* = -0.31), and the strongest negative correlation with IFN-γ (*r* = -0.53).

## 4 Discussion

In recent years, the role of the tryptophan metabolic pathway in disease regulation has garnered increasing attention (Chen et al., 2024), with research primarily focused on neuropsychiatric disorders, digestive diseases, immune regulation, and oncology, particularly investigating depression, inflammatory bowel disease, metabolic disorders, and malignancies (Correia and Vale, 2022; Davidson et al., 2022; Peyraud et al., 2022). However, its implications in viral infections, especially HBV infection, remain relatively underexplored.

This study conducted a retrospective analysis of 106 CHB patients who received NAs therapy for over 48 weeks and 29 healthy controls, aiming to explore the relationship between tryptophan metabolites and cytokines with HBV treatment outcomes. The study found that multiple tryptophan metabolic pathways were closely associated with HBV treatment efficacy, particularly IAld in the bacterial degradation pathway, which showed a remarkable significant gradient correlation with treatment response and a negative correlation with HBsAg titer, a finding not previously reported in previous studies. Additionally, several metabolites in the 5-HT pathway and the kynurenine pathway also exhibited significant intergroup variations. These findings not only reveal the potential role of tryptophan metabolism in HBV treatment but also provide new perspectives and potential targets for future research and clinical applications.

In this study, the bacterial degradation pathway metabolite IAld was significantly higher in the cured group than in the non-cured group, and showed significant correlations with HBsAg levels, IFN-γ, IL-4, IL-1β, IL-17, IL-2, and TNF-α, suggesting that IAld may play an important role in the immune regulation of HBV antigen clearance. The bacterial degradation pathway is mediated by the gut microbiota, with some commensal microbiota producing AhR ligands, which generate IAA and IPA through oxidative and reductive pathways, thereby influencing intestinal permeability and host immunity (Dodd et al., 2017). AhR is a ligand-dependent transcription factor activated by various synthetic or biomolecular compounds, playing a crucial role in immune responses and inflammation suppression (Stockinger et al., 2014). IAld has recently been identified as an AhR (Zelante et al., 2013), with studies demonstrating that microbiota-derived Trp metabolites like IAld play protective roles against mucosal candidiasis (Zelante et al., 2013). In dextran sodium sulfate (DSS)-induced mouse colitis, dietary Trp supplementation restores AhR ligands produced by the gut microbiota, thereby alleviating the severity of colitis (Islam et al., 2017). Similarly, in experimental autoimmune encephalomyelitis models, supplementation with Trp or Trp-derived AhR agonists enhanced IFN suppression and limit central nervous system (CNS) inflammation in an AhR-dependent mechanisms (Rothhammer et al., 2016). Recent studies have also found that IAld can ameliorate Coronavirus disease 2019 (COVID-19)-associated pulmonary aspergillosis (CAPA) during Severe Acute Respiratory Syndrome Coronavirus 2 (SARS-CoV-2) and A. fumigatus co-infection by multiple mechanisms, including protecting the epithelial barrier, restoring virus-induced pro-inflammatory features by reducing IL-1β production and increasing IL-10 to promote antiviral responses and limit viral replication, and IAld works better in a prophylactic rather than therapeutic protocol (Pariano et al., 2024). Unlike the aforementioned study, all disease groups in this study exhibited decreased IL-1β and increased IL-10 levels, which is consistent with the conclusions of other studies. The studies have found that in CHB patients non-responsive to IFN-α therapy, IL-1β levels may be significantly suppressed, possibly due to inhibition of NF-κB signaling and activation of the inflammasome/caspase-1 pathway (Lei et al., 2019; Wang et al., 2019). Additional research has found that stimulation of HBV-infected cells with IL-1β *in vitro* can rapidly reduce intracellular HBV RNA levels (Delphin et al., 2021). These studies suggest that HBV may maintain persistent infection by inhibiting the antiviral effects of IL-1β, while IL-1β may serve as a potential therapeutic candidate molecule against HBV. Based on these studies and the results of this study, we may also hypothesize that supplementation or stimulation of IAld and IL-1β could regulate the HBV immune response and promote the clearance of HBsAg, though the conclusions and potential mechanisms require further investigation.

Our study found that multiple 5-HT pathway metabolites were significantly lower in non-cured patients compared to both cured patients and healthy controls, suggesting a potential association with 5-HT’s anti-inflammatory role in immune regulation. 5-HT, also known as serotonin, is produced in the central nervous system, gastrointestinal tract and platelets (Brenner et al., 2007). It functions as both a neurotransmitter and peripheral hormone, regulating diverse physiological and psychological processes including appetite, sleep, pain, and mood. Through binding to 5-HT receptors on immune cells, it also serves as an effective regulator of both the innate and adaptive immune systems. A recent study on post-acute sequelae of COVID-19 (PASC, or “Long COVID”) found that PASC is associated with reduced serotonin levels, and other acute viral infections also cause a significant serotonin depletion. Although serotonin levels can rebound to baseline after acute infection resolution, chronic viral infections may cause persistent serotonin deficiency—consistent with our findings. The study also suggests that viral infections and type I interferon-driven inflammation reduce serotonin levels through diminished intestinal absorption of the serotonin precursor tryptophan, platelet overactivation and thrombocytopenia and monoamine oxidase (MAO)-mediated serotonin turnover (Wong et al., 2023). Similarly, Wu et al. also found that serotonin levels were significantly lower in decompensated CHB patients than in compensated CHB patients, and serotonin levels were lower in the HCC group than in the CHB group regardless of HBV DNA levels, suggesting serotonin may be a good prognostic marker (Wu et al., 2022). However, increasing evidence also suggests 5-HT’s contradictory role in immunomodulatory, exerting both stimulatory and inhibitory activities (Mawe and Hoffman, 2013; Renga et al., 2023). Earlier studies have also suggested that serotonin acts as a chemokine to increase the secretion of pro-inflammatory cytokines (IL-1, IL-6, NF-κB) and enhance phagocytosis (Kanova and Kohout, 2021). In patients with inflammatory bowel disease (IBD), 5-HT also increases Nicotinamide adenine dinucleotide phosphate (NADPH) -dependent reactive oxygen species (ROS) production and upregulates IL-6 and IL-8 (Regmi et al., 2014). And serotonin stored in platelets has been shown to supports viral persistence in the liver and exacerbates cytotoxic T lymphocyte (CTL) -mediated liver immunopathology in viral hepatitis (Lang et al., 2008). Although these studies suggest that 5-HT may have pro-inflammatory effects in certain inflammatory diseases, the causal relationship and specific mechanisms between 5-HT and treatment outcomes in HBV patients require further exploration.

IDO, which catalyzes the conversion of tryptophan to kynurenine, has emerged as a prominent research focus due to its dual immunoregulatory roles. This enzyme can be induced by pro-inflammatory cytokines including IFN-γ and IL-6 (Werner et al., 1987; Xie et al., 2023), promotes immune tolerance by T-cell suppression, and simultaneously inhibiting replication of intracellular pathogens such as human immunodeficiency virus (HIV), herpes simplex virus (HSV), or cytomegalovirus (CMV) (Adams et al., 2004; Boasso and Shearer, 2007; Cassoux et al., 1999). Studies suggest IDO may exert anti-HBV effects through reciprocal activation of NK cells and pDCs, mediated by IFN-γ and IFN-α release. In patients with acute hepatitis B, early strong activation of IDO is a marker of successful clearance of HBV (Yoshio et al., 2016). Mao et al. also found that IFN-γ-induced IDO can inhibit HBV replication in HepG2 cells transfected with the HBV genome (Mao et al., 2011). Our findings of elevated Kyn/Trp ratios in cured patients align with previous reports, though we observed particularly high levels in HBeAg-positive group. Through a comprehensive literature review, we identified that Kyn, produced by IDO1 through the metabolism of Trp, serves as an endogenous ligand for the AhR. Binding of Kyn to AhR induces the differentiation of immature CD4+ T cells into regulatory T cells. Additionally, Kyn binding to AhR can also induce IDO1 expression, further suppressing T cell immune responses (Proietti et al., 2020). Previous studies have also suggested that kynurenine produced by IDO exacerbates liver damage in HBV specific CTL-induced fulminant hepatitis (Ohtaki et al., 2014). Similar inhibitory effects have been observed in HIV, the serum kynurenine-to-tryptophan ratio in HIV-infected patients increases with disease progression and immune stimulation (Huengsberg et al., 1998). Additionally, studies have shown that the proportion of granulocytic myeloid-derived suppressor cells (gMDSCs) in peripheral blood and liver tissue of HBV-ACLF patients significantly increases and inhibits T cell proliferation and IFN-γ production via IDO/IL-10 pathway (Yu et al., 2022). HBeAg-positive patients exhibit higher circulating MDSC frequencies than HBeAg-negative individuals, as HBeAg induce the expansion of MDSCs by upregulating IDO, thereby impairing T-cell function and maintaining HBV persistent infection, which is consistent with our findings (Yang et al., 2019). Increased IDO activity directly or indirectly influences the production of various cytokines, including IL-10, IFN-γ, and TNF-α, thereby participating in the regulation of inflammatory responses and immune tolerance (de Araújo et al., 2017; Huang et al., 2020). IL-10, as a potent anti-inflammatory cytokine, may also suppress the activity of Thelper type 1 (Th1) cells, Natural Killer (NK) cells, and macrophages to impede pathogen clearance (Couper et al., 2008). While our data show no significant correlation between Kyn/Trp and cytokines like TNF-α, IL-10, and IL-6, their coordinated directional changes suggest IDO may influence multiple cytokine networks in HBeAg-positive patients, potentially hindering HBeAg clearance. These findings collectively position IDO as a double-edged sword in HBV-related inflammation (Munn and Mellor, 2013; Xu et al., 2008), warranting cautious consideration of IDO as a potential therapeutic target for HBV infection.

In the kynurenine pathway, we also found that 3-HK/Kyn ratios (reflecting KMO activity), was elevated in non-cured groups, while downstream metabolites including KA (KAT product), XA/3-HK (KAT II activity), 3-HAA/3-HK (KYNU activity), and the terminal metabolite NAA were all reduced across patient groups. These findings suggest a potential metabolic blockade downstream of 3-HK in nucleos(t)ide analogue-treated HBV patients, particularly those failing to achieve functional cure. This metabolic profile is similar to the tryptophan metabolite patterns observed by Moulin et al. in rheumatoid arthritis (Moulin et al., 2024), while the use of exogenous catalysts conversion of Kyn and 3-HK to produce XA and KA via the recombinant enzyme aminoadipate aminotransferase (AADAT) directly regulates endogenous tryptophan metabolism through AhR activation and cellular metabolic reprogramming, thereby reducing the severity of inflammatory bowel disease and rheumatoid arthritis (Michaudel et al., 2023; Moulin et al., 2024). Therefore, regulating the kynurenine pathway may also become a new therapeutic strategy for HBV infection.

There were also some interesting findings in this study. The PA concentration and PA/3-HAA ratio were exclusively elevated in the cured group, showing significant increases compared to both non-cured patients and healthy controls. And these measurements demonstrated negative correlations with HBsAg and IL-2 levels, while exhibiting positive correlations with cytokines including IL-1β, IL-5, IL-10, and TNF-α, potentially reflecting unique regulatory mechanisms in the terminal stages of tryptophan metabolism among cured individuals. PA is considered a naturally toxic pyridine derivative and an important intermediate widely used in the chemical industry (Shi et al., 2024; Xu et al., 2022; Yan et al., 2023). In the field of biological research, picolinic acid and its derivatives have been shown to possess antibacterial and antifungal properties (Tamer et al., 2018). Research indicates PA may be involved in macrophage activation in the human body (Melillo et al., 1996), and exhibit neuroprotective and osteoprotective effects (Duque et al., 2020; Knapskog et al., 2023). Notably, recent studies have also revealed PA’s broad-spectrum antiviral activity against enveloped viruses - including SARS-CoV-2, influenza A virus (IAV), flaviviruses, HSV, and paramyxoviruses - through mechanisms involving viral membrane integrity disruption, fusion inhibition of virus-cell membrane, and cellular endocytosis interference, while it is ineffective against non-enveloped viruses and bacteriophages. Therefore, PA is also an important target for broad-spectrum antiviral drugs (Narayan et al., 2023). However, high-dose PA (≥ 500 mg/kg/d) may induce neurovascular toxicity (Knapskog et al., 2023). We reasonably hypothesize that elevated PA levels in cured group may contribute significantly to HBV clearance. Whether PA also exhibits antiviral properties in the enveloped virus HBV is a question worthy of further exploration, which also provides a new perspective on PA as a therapeutic target for HBV.

This study still has some limitations. First, the relatively small sample size may have affected the statistical power of our findings. Second, none of the participants in this study had received interferon therapy. With the WHO’ s strategy to eliminate hepatitis B as a public health threat by 2030 in 2016 (World Health Organization, 2016), the application of interferon therapy in the treatment of HBV infection has gradually expanded. The complex regulatory effects of IFN-α on tryptophan metabolism further underscore this limitation. Additionally, while this study did not delve into the specific mechanisms of tryptophan metabolic pathway in HBV treatment regulation, our preliminary findings may provide direction for future research. Subsequent studies should incorporate larger cohorts including interferon-treated patients to validate the role of tryptophan metabolism across different therapeutic regimens. Furthermore, in-depth research into the interaction mechanisms between the tryptophan metabolic pathway and the immune system, along with clinical prospective studies, such as the specific role of IAld in HBV immune regulation, could help elucidate the potential application of tryptophan metabolites as biomarkers or therapeutic targets in HBV therapy or for identifying patient populations with higher likelihood of functional cure.

In summary, this study represents the first systematic analysis of tryptophan metabolic pathway alterations in chronic HBV infection, establishing a correlation map across tryptophan metabolic profiles, cytokines and HBV functional cure under NAs therapy. The findings revealed that functional cure patients exhibited distinct metabolic characteristics: IAld levels showed negative correlations with HBsAg titer and pro-inflammatory cytokine IFN-γ, while demonstrating positive correlations with pro-inflammatory cytokine IL-1β. Elevated PA levels were associated with decreased IL-2 and increased IL-1β and IL-10, and higher 5-HT levels were accompanied by increased IL-1β and IFN-α. These results not only uncover the coordinated regulatory network between tryptophan metabolites (IAld, PA, 5-HT, etc.) and key cytokines (IL-1β, IL-10, IFN-γ, etc.), providing new perspectives for immunometabolic research in viral infections, but also establish a theoretical foundation for developing biomarkers for HBV treatment by identifying characteristic association patterns between metabolites and cytokines, offering important insights for designing therapeutic strategies targeting immunometabolic pathways. Targeting Trp metabolites represents a novel and promising strategy (Xue et al., 2023). The strong associations of IAld and PA with functional cure, alongside their established immunomodulatory roles in viral infections, suggest these metabolites as candidate therapeutic nodes. A comprehensive investigation of the role and regulatory mechanisms of Trp metabolites and immune in HBV treatment is warranted to facilitate their clinical application.

## Data Availability

The original contributions presented in the study are included in the article/**Supplementary Material**. Further inquiries can be directed to the corresponding authors.

## Supplementary material

Supplementary Figure 1 Tryptophan metabolic pathway.

Supplementary Figure 2 Principal component analysis of tryptophan metabolites and cytokines.

Supplementary Figure 3 Principal component analysis of tryptophan metabolites and cytokines across different groups.

Supplementary Table 1 Comparison of clinical laboratory indicators between functional cure group and non-cured groups.

Supplementary Table 2 The levels of tryptophan metabolites and cytokines in each group.
