# Supplementary material for: Tryptophan metabolic reprogramming modulates cytokine networks in nucleos(t)ide analogue-treated chronic hepatitis B patients
Source: Front Cell Infect Microbiol. 2025 Jul 21;15:1643636. doi: 10.3389/fcimb.2025.1643636 (PMC12319034; doi:10.3389/fcimb.2025.1643636)
Supplement: Supplementary Figure 1 — Tryptophan metabolic pathway. [file DataSheet1.zip › Supplementary Materials Correction/Supplementary Materials Correction.docx]

Supplementary Materials

# Supplementary Figures


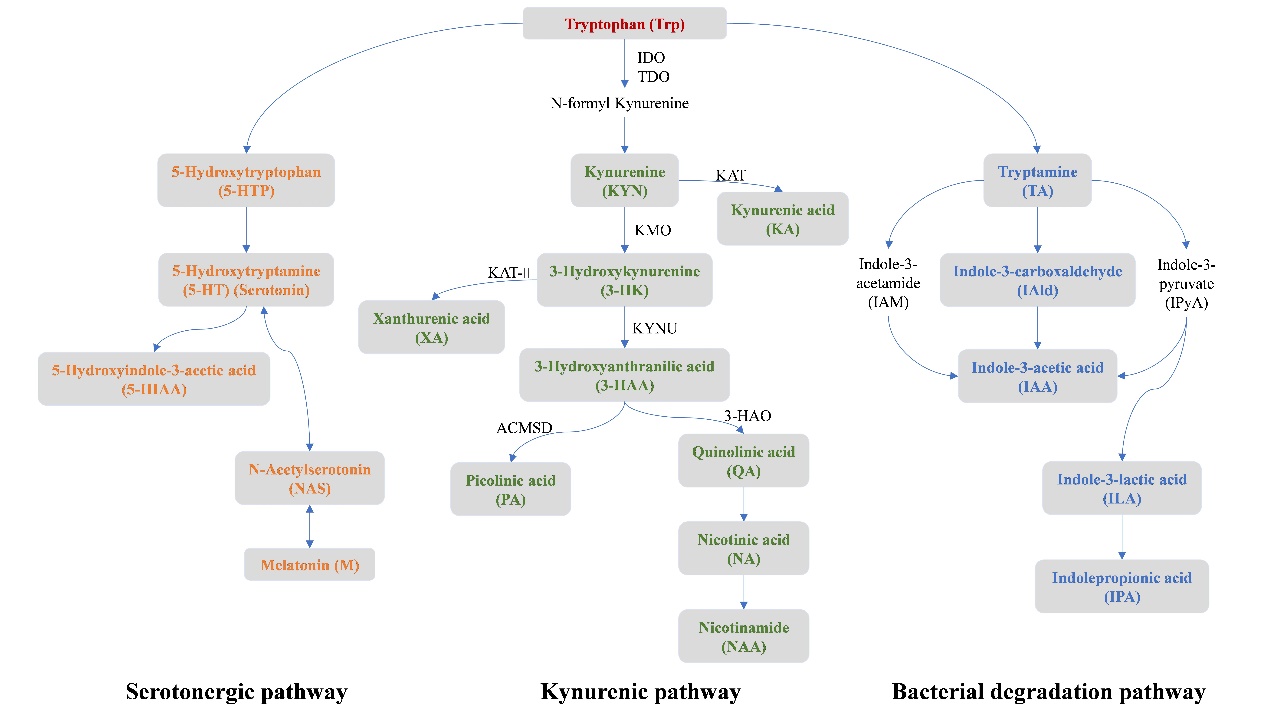


Supplementary Figure 1. Tryptophan metabolic pathway. The tryptophan and its metabolites detected in this study are highlighted with gray background. Abbreviations: IDO, Indoleamine 2,3-dioxygenase; TDO, Tryptophan 2,3-dioxygenase; KAT, kynurenine aminotransferase; KMO, kynurenine 3-monooxygenas; KYNU, kynureninase; ACMSD, aminocarboxymuconate semialdehyde decarboxylase; 3-HAO, 3-hydroxyanthranilate 3,4-dioxygenase.


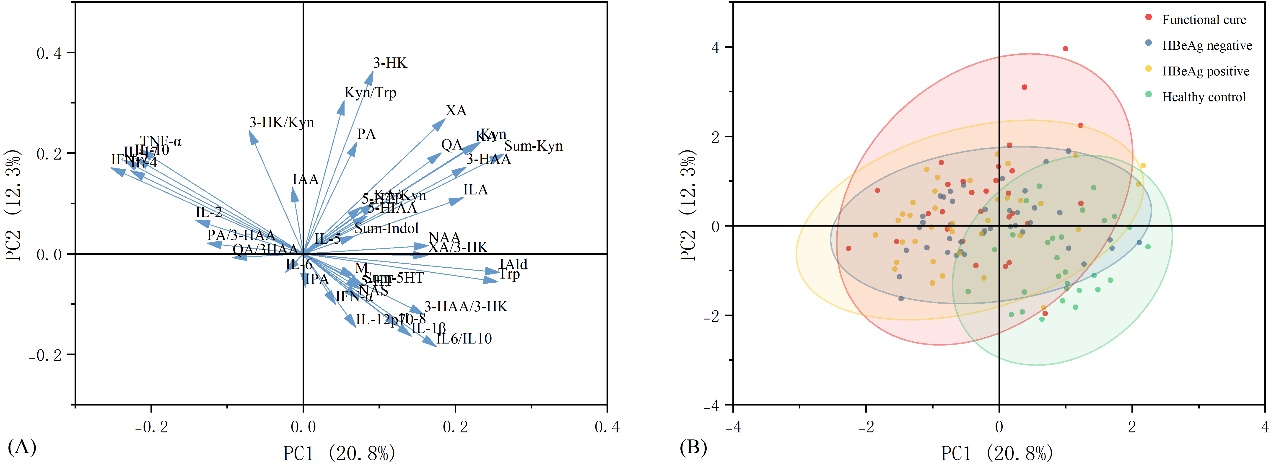


Supplementary Figure 2. Principal component analysis of tryptophan metabolites and cytokines. (A) Loading plot: metabolites and cytokines contributing to PC1 and PC2 are shown. Arrow length and direction represent their correlation with principal components. (B) Score plot: Samples are projected onto PC1 and PC2. Samples are colored by treatment outcome: functional cure group (red, n = 29), HBeAg-negative group (blue, n = 40), HBeAg-positive group (yellow, n = 37), and healthy controls group (green, n = 29). Ellipses represent 95% confidence intervals for each group. PC1 and PC2 explained 20.8% and 12.3% of the total variance, respectively.


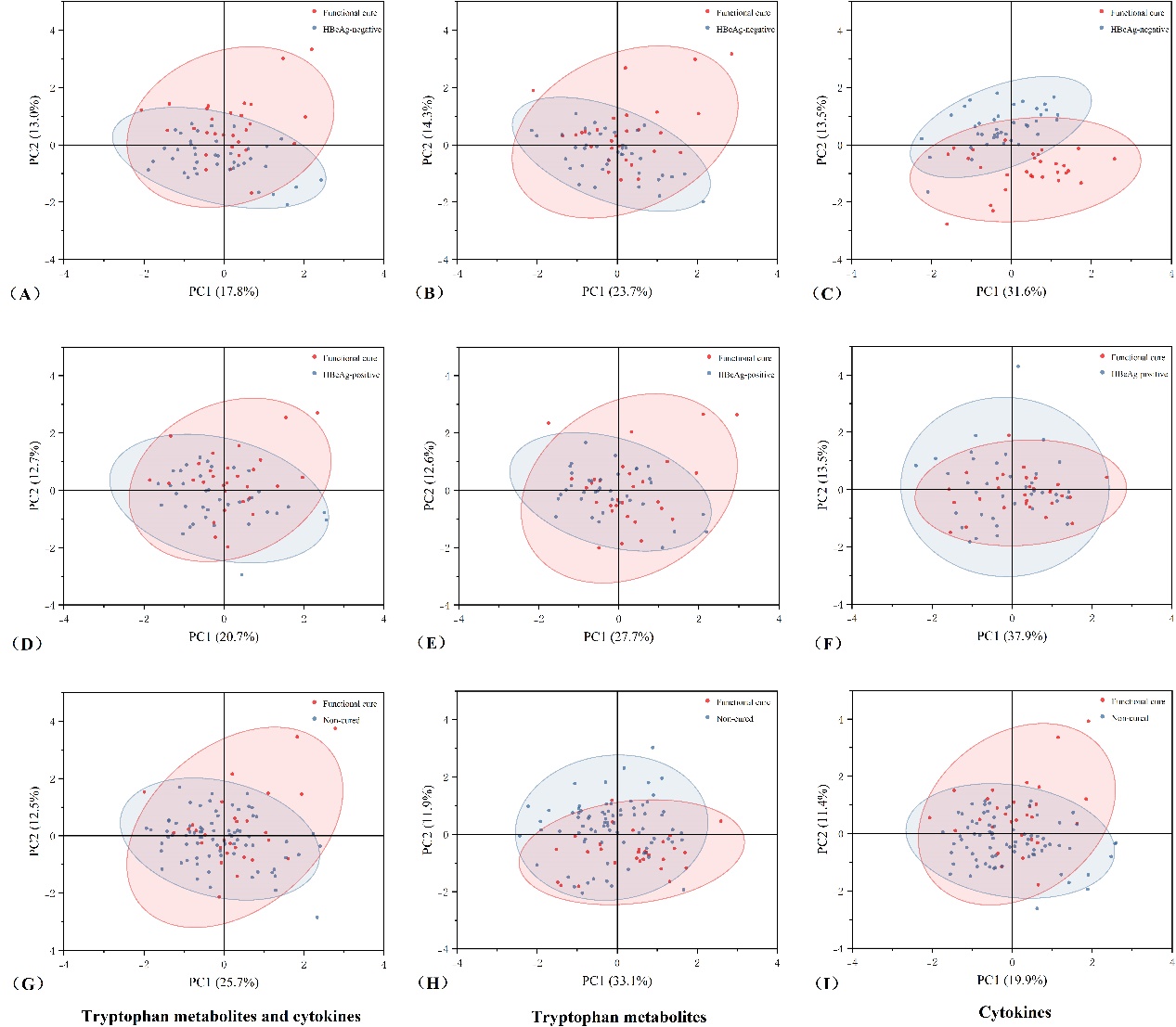


Supplementary Figure 3. Principal component analysis of tryptophan metabolites and cytokines across different groups. (A-C) PCA for functional cure (n = 29) versus HBeAg-negative (n = 40), (D-F) PCA for functional cure (n = 29) versus HBeAg-positive (n = 37), and (G-I) PCA for functional cure (n = 29) versus non-cured groups (combined HBeAg-negative and HBeAg-positive) (n = 77). (A/D/G) PCA of Tryptophan Metabolites and Cytokines, (B/E/H) PCA of Tryptophan Metabolites, (C/F/I) PCA of Cytokines.

# Supplementary Tables

**Supplementary Table 1.** **Comparison of clinical laboratory indicators between functional cure group and non-cured groups**

| Groups | Functional cure group  (n = 29) | Non-cured group  (n = 77) | *P* value^a^ |
| --- | --- | --- | --- |
| ALT (U/L) | 26 (15.75, 35) | 22 (16, 30) | 0.161 |
| AST (U/L) | 23.5 (21, 25.75) | 23 (20, 26) | 0.628 |
| ALP (IU/L) | 83.81 ± 16.38 | 81.28 ± 22.87 | 0.480 |
| GGT (IU/L) | 21.5 (13.75, 28.25) | 18 (12.25, 27) | 0.318 |
| ALB (g/L) | 48.50 ± 2.39 | 47.62 ± 2.34 | 0.092 |
| WBC (×10^9^/L) | 5.84 ± 1.62 | 5.91 ± 1.37 | 0.636 |
| HBV DNA (n (%)) |  |  |  |
| <100 copies/mL | 29 (100%) | 73 (89.2%) | 0.211 |
| ≥100 copies/mL |  | 4 (10.8%) |  |

a. Analysis of inter-group differences was conducted by Mann-Whitney U test for ALT, AST, GGT, Student T test for ALP, ALB, WBC and Pearson Chi-square for HBV DNA.

Abbreviation: ALT, Alanine Aminotransferase; AST, Aspartate Aminotransferase; ALP, Alkaline Phosphatase; GGT, Gamma-Glutamyl Transferase; ALB, Albumin; WBC, White Blood Cell Count.

**Supplementary Table 2.** **The levels of tryptophan metabolites and cytokines in each group**

| **Group^a^** | **Functional cure (n = 29)** | **HBeAg-negative (n = 40)** | **HBeAg-positive (n = 37)** | **Healthy Control (n = 29)** | ***P* value^b^** |
| --- | --- | --- | --- | --- | --- |
| Trp (μmol/L) | 48.342 (43.123, 55.091) | 49.141 (44.625, 57.709) | 44.136 (39.683, 51.749) | 56.021 (50.214, 61.151) | < 0.001 |
| Sum-5-HT (nmol/L) | 106.871 (74.326, 285.95) | 108.477 (68.41, 182.333) | 72.62 (54.845, 108.563) | 177.103 (89.962, 436.207) | < 0.001 |
| 5-HTP (nmol/L) | 2.913 (2.463, 3.242) | 2.814 (2.452, 3.245) | 2.562 (2.37, 3.216) | 2.544 (2.252, 3.181) | 0.395 |
| 5-HT (nmol/L) | 67.495 (34.3, 246.712) | 69.542 (34.29, 144.908) | 39.841 (23.965, 69.541) | 134.806 (56.103, 398.365) | < 0.001 |
| 5-HIAA (nmol/L) | 33.308 (28.461, 40.656) | 32.857 (27.977, 38.574) | 29.172 (25.831, 34.477) | 34.204 (28.515, 39.928) | 0.233 |
| NAS (nmol/L) | 0.054 (0.032, 0.096) | 0.033 (0.021, 0.045) | 0.036 (0.031, 0.053) | 0.059 (0.03, 0.08) | 0.008 |
| M (nmol/L) | 0.013 (0.008, 0.021) | 0.013 (0.01, 0.03) | 0.016 (0.01, 0.024) | 0.031 (0.019, 0.047) | 0.001 |
| Sum-Indoles (nmol/L) | 3688.685 (2677.209,5887.274) | 3206.125 (2522.87, 3935.4) | 3613.84(2692.059, 4834.025) | 4359.325 (3136.518, 5099.104) | 0.059 |
| IAld (nmol/L) | 91.44 (71.489, 123.58) | 63.324 (44.6, 82.175) | 65.938 (42.016, 88.04) | 130.634 (108.649, 156.43) | < 0.001 |
| IAA (nmol/L) | 1722.251 (1140.779, 3011.986) | 1412.512 (1059.968, 2013.478) | 1581.037 (1081.151, 2423.417) | 1617.86 (1240.689, 1847.429) | 0.547 |
| ILA (nmol/L) | 720.598 (671.643, 933.161) | 682.674 (540.002, 962.435) | 698.231 (573.46, 906.926) | 931.795 (794.953, 1073.589) | 0.001 |
| IPA (nmol/L) | 757.865 (300.921, 2216.464) | 767.841 (473.726, 1515.791) | 794.786 (554.738, 1407.173) | 1090.491 (630.409, 2214.835) | 0.365 |
| Sum-Kyn (nmol/L) | 2691.723 (2377.415, 3100.436) | 2441.405 (2171.359, 2930.095) | 2392.473 (2043.508, 2808.963) | 2717.804 (2518.255, 3056.65) | 0.014 |
| Kyn (nmol/L) | 1742.739 (1523.117, 2088.743) | 1569.232 (1364.495, 1922.225) | 1623.745 (1378.701, 1899.229) | 1808.944 (1509.159, 1934.27) | 0.068 |
| KA (nmol/L) | 62.902 (48.922, 73.613) | 59.632 (46.845, 67.428) | 47.669 (40.626, 66.026) | 65.536 (53.716, 81.315) | 0.024 |
| 3-HK (nmol/L) | 30.89 (27.172, 37.924) | 33.114 (27.493, 41.834) | 29.229 (23.527, 41.586) | 29.701 (23.073, 36.344) | 0.322 |
| XA (nmol/L) | 14.821 (12.512, 21.152) | 13.867 (10.077, 19.622) | 12.509 (8.509, 17.605) | 16.054 (10.824, 24.255) | 0.17 |
| 3-HAA (nmol/L) | 10.558 (8.597, 14.325) | 12.571 (10.531, 15.935) | 9.444 (8.15, 12.661) | 12.166 (9.549, 16.981) | 0.058 |
| PA (nmol/L) | 44.14 (36.556, 56.198) | 26.696 (23.462, 36.078) | 28.666 (25.239, 36.94) | 31.507 (22.432, 37.918) | < 0.001 |
| QA (nmol/L) | 279.726 (251.947, 325.255) | 287.076 (241.798, 339.159) | 235.792 (200.088, 332.742) | 295.143 (237.15, 370.551) | 0.083 |
| NAA (nmol/L) | 407.086 (325.487, 489.324) | 425.692 (325.949, 487.036) | 385.517 (282.006, 431.75) | 503.661 (390.488, 648.359) | 0.009 |
| Kyn/Trp | 36.94 (32.627, 41.018) | 32.496 (29.163, 34.391) | 36.958 (30.366, 42.675) | 31.434 (28.88, 36.084) | < 0.001 |
| KA/Kyn | 0.033 (0.03, 0.038) | 0.035 (0.032, 0.042) | 0.032 (0.026, 0.039) | 0.038 (0.03, 0.044) | 0.085 |
| 3-HK/Kyn | 0.018 (0.015, 0.021) | 0.02 (0.017, 0.025) | 0.019 (0.017, 0.022) | 0.015 (0.013, 0.02) | 0.004 |
| XA/3-HK | 0.497 (0.397, 0.59) | 0.451 (0.336, 0.547) | 0.447 (0.329, 0.554) | 0.578 (0.519, 0.659) | 0.001 |
| 3-HAA/3-HK | 0.324 (0.267, 0.449) | 0.379 (0.29, 0.486) | 0.373 (0.277, 0.421) | 0.424 (0.347, 0.559) | 0.027 |
| QA/3-HAA | 26.941 (22.384, 30.839) | 22.013 (17.978, 28.642) | 24.277 (21.897, 29.223) | 23.908 (20.295, 29.426) | 0.174 |
| PA/3-HAA | 4.243 (3.211, 5.25) | 2.179 (1.834, 2.665) | 3.132 (2.064, 3.852) | 2.391 (1.931, 3.527) | < 0.001 |
| IL-2 (pg/mL) | 1.48 (1.055, 1.845) | 2.88 (2.298, 3.865) | 1.65 (1.04, 2.02) | 1.17 (0.455, 2.705) | < 0.001 |
| IL-4 (pg/mL) | 1.49 (1.205, 1.955) | 1.44 (1.06, 1.61) | 1.13 (0.815, 1.75) | 0.5 (0.25, 1.33) | < 0.001 |
| IL-6 (pg/mL) | 2.86 (2.135, 3.525) | 2.35 (1.848, 2.963) | 2.56 (1.925, 3.45) | 2.61 (2.155, 3.44) | 0.039 |
| IL-10 (pg/mL) | 5.9 (4.55, 7.065) | 3.21 (2.36, 4.448) | 4.15 (3.245, 6.34) | 2.24 (1.57, 3.26) | < 0.001 |
| TNF-α (pg/mL) | 5.22 (2.925, 6.03) | 1.895 (1.495, 2.43) | 2.67 (1.655, 5.21) | 0.68 (0.215, 1.74) | < 0.001 |
| IFN-γ (pg/mL) | 4.84 (3.48, 6.005) | 4.62 (3.808, 5.76) | 4.57 (3.015, 5.635) | 2.01 (0.995, 4.045) | < 0.001 |
| IL-17 (pg/mL) | 5.68 (3.975, 6.19) | 4.63 (3.55, 5.89) | 4.3 (3.645, 6.06) | 2.22 (1.425, 4.465) | < 0.001 |
| IL-1β (pg/mL) | 0.94 (0.465, 1.7) | 0.9 (0.465, 1.48) | 0.68 (0.265, 1.2) | 3.76 (2.41, 6.855) | < 0.001 |
| IL-5 (pg/mL) | 0.78 (0.46, 1.005) | 0.65 (0.37, 0.88) | 0.71 (0.31, 1.13) | 0.78 (0.513, 1.17) | 0.472 |
| IL-12p70 (pg/mL) | 0.9 (0.525, 1.18) | 1.1 (0.72, 1.52) | 0.875 (0.245, 1.145) | 1.56 (1.28, 2.18) | < 0.001 |
| IFN-α (pg/mL) | 0.76 (0.1, 1.05) | 0.95 (0.413, 1.28) | 1.09 (0.22, 1.57) | 1.56 (0.8, 2.325) | 0.006 |
| IL-8 (pg/mL) | 10.335 (8.395, 11. 873) | 7.855 (6.55, 11.063) | 8.16 (6.503, 10.153) | 13.83 (11.305, 20.89) | < 0.001 |
| IL-6/IL-10 | 0.51 (0.35, 0.695) | 0.69 (0.48, 1.02) | 0.58 (0.415, 0.855) | 1.37 (0.845, 1.605) | < 0.001 |

a. Functional cure: HBV-infected patients with sustained HBsAg loss and undetectable HBV DNA; HBeAg-negative: patients with HBsAg+HBeAg-HBcAb+; HBeAg-positive: patients with HBsAg+HBeAg+HBcAb+.

b. *P* value < 0.05 indicates statistical significance.

Abbreviation: Trp, Tryptophan; Sum-5-HT, Collective metabolites in the serotonin pathway; 5-HTP, 5-Hydroxytryptophan; 5-HT, 5-Hydroxytryptamine (Serotonin); 5-HIAA, 5-Hydroxyindoleacetic acid; NAS, N-Acetylserotonin; M, Melatonin; Sum-Indol, Collective metabolites in the bacterial degradation pathway (Indole pathway); IAlD, Indole-3-carboxaldehyde; IAA, Indole-3-acetic acid; ILA, Indole-3-lactic acid; IPA, Indolepropionic acid; Sum-Kyn, Collective metabolites in the kynurenine pathway; Kyn, Kynurenine; KA, Kynurenic acid; 3-HK, 3-Hydroxykynurenine; XA, Xanthurenic acid; 3-HAA, 3-Hydroxyanthranilic acid; PA, Picolinic acid; QA, Quinolinic acid; NAA, Nicotinic acid adenine dinucleotide; IL, Interleukin; TNF, Tumor Necrosis Factor; IFN, Interferon.
