# Supplementary figures and images for: Tryptophan metabolic reprogramming modulates cytokine networks in nucleos(t)ide analogue-treated chronic hepatitis B patients
Source: Front Cell Infect Microbiol. 2025 Jul 21;15:1643636. doi: 10.3389/fcimb.2025.1643636 (PMC12319034; doi:10.3389/fcimb.2025.1643636)

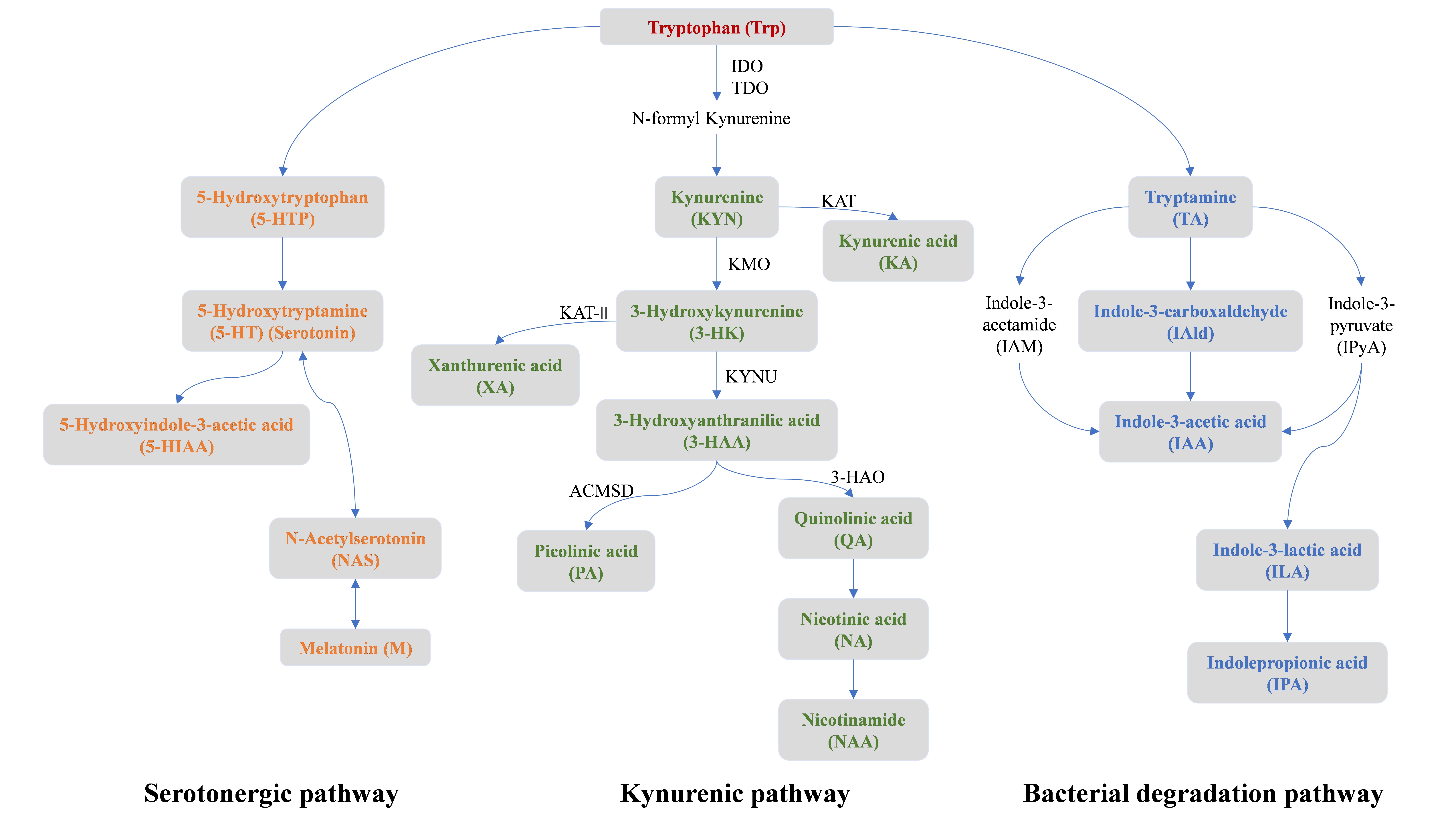

Supplement: Supplementary Figure 1 — Tryptophan metabolic pathway. [file DataSheet1.zip › Supplementary Materials Correction/Supplementary Figure 1.jpg]

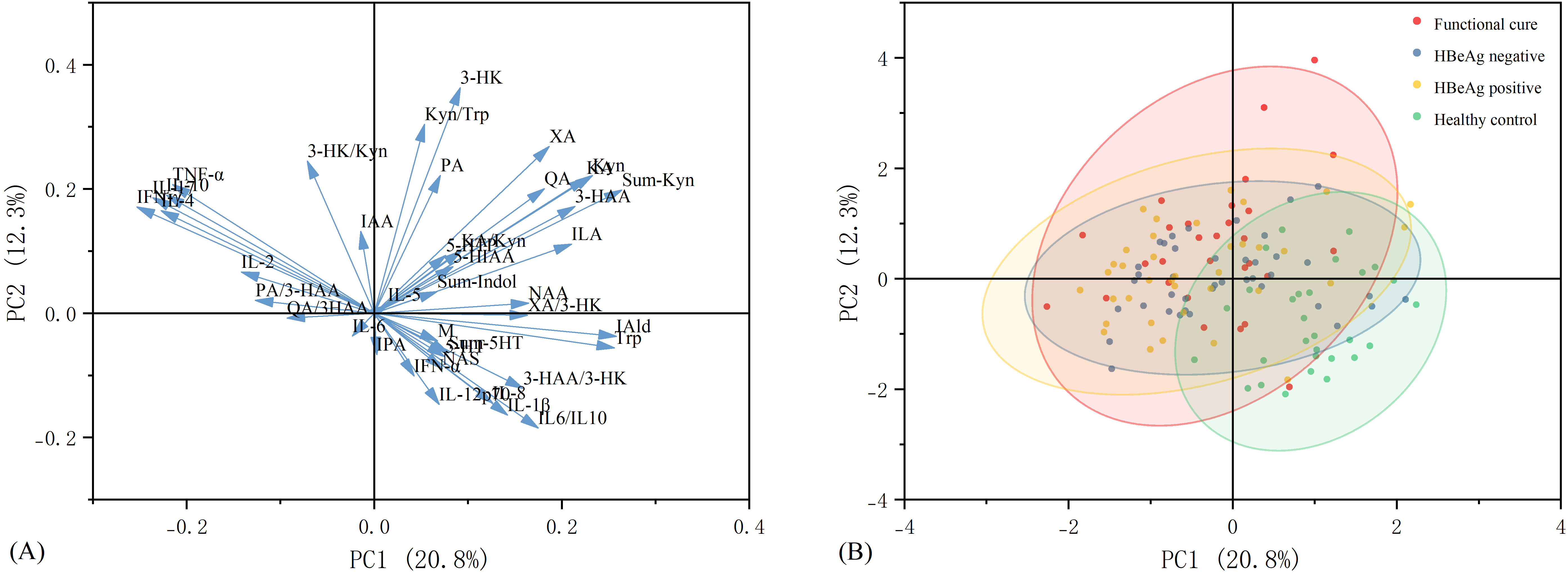

Supplement: Supplementary Figure 1 — Tryptophan metabolic pathway. [file DataSheet1.zip › Supplementary Materials Correction/Supplementary Figure 2.jpg]

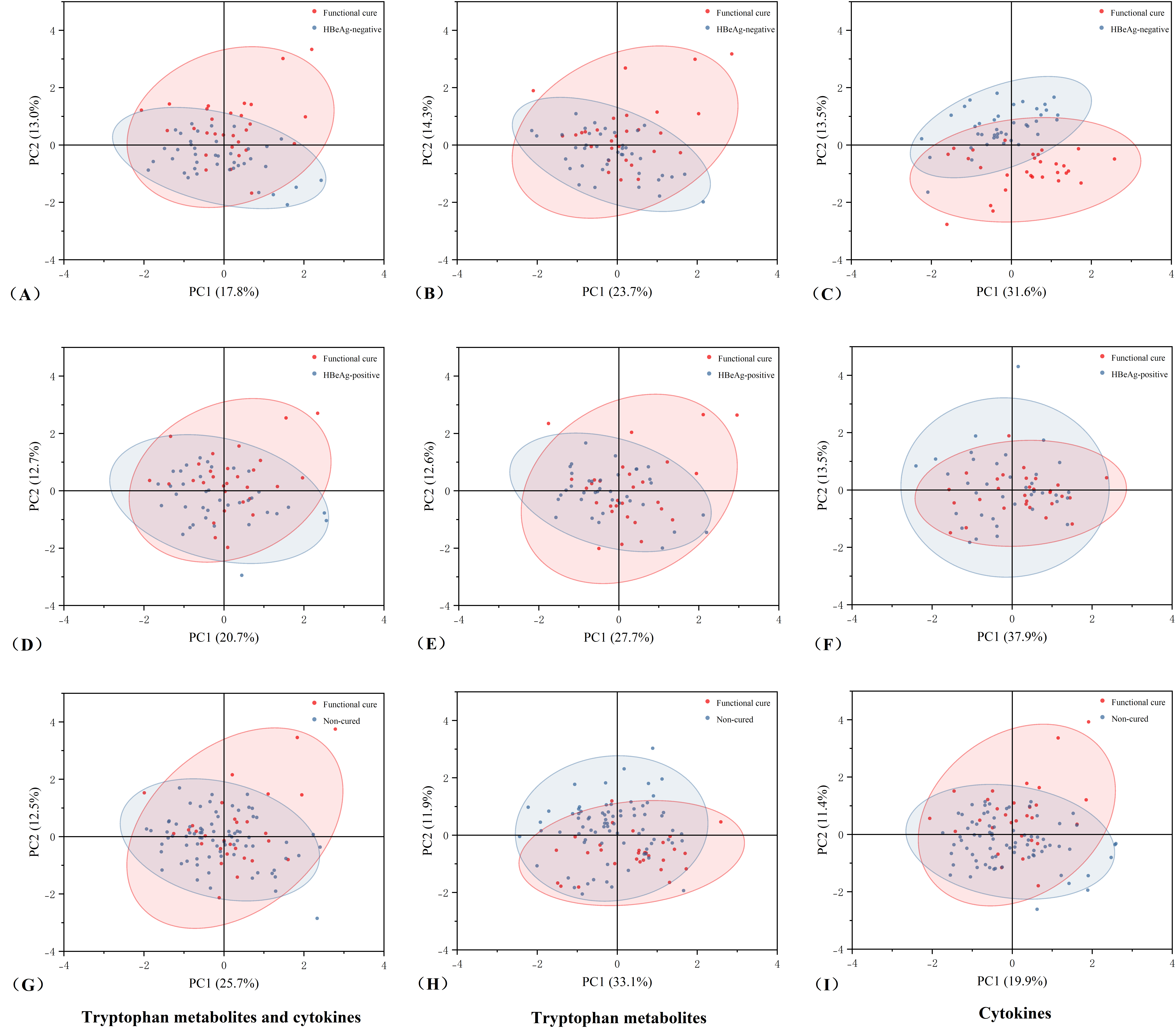

Supplement: Supplementary Figure 1 — Tryptophan metabolic pathway. [file DataSheet1.zip › Supplementary Materials Correction/Supplementary Figure 3.jpg]
